# Optimization of Extraction Conditions for Improving Gallic Acid and Quercetin Content in *Pouteria macrophylla* Fruits: A Promising Cosmetic Ingredient

**DOI:** 10.1021/acsomega.4c11241

**Published:** 2025-02-13

**Authors:** Camila F. B. Albuquerque, Dayenne A. A. de Souza, Pablo Luis B. Figueiredo, Cláudia Quintino Rocha, José Guilherme S. Maia, Massuo J. Kato, Renan Campos Chisté, Joyce Kelly R. da Silva

**Affiliations:** † Programa de Pós-Graduação em Biotecnologia, Instituto de Ciências Biológicas, Universidade Federal do Pará, Belém, PA 66075-110, Brazil; ‡ Programa de Pós-Graduação em Química, Instituto de Química, Universidade Federal do Pará, Belém, PA 66075-110, Brazil; § Laboratório de Química dos Produtos Naturais, Universidade do Estado do Pará, Belém, PA 66095-015, Brazil; ∥ Programa de Pós-Graduação em Química, Universidade Federal do Maranhão, São Luís, MA 65085-580, Brazil; ⊥ Laboratório de Química de Produtos Naturais, Instituto de Química, Universidade de São Paulo, São Paulo, SP 05508-000, Brazil; # Faculdade de Farmácia, Universidade Federal de Minas Gerais, Belo Horizonte, MG 31270-901, Brazil

## Abstract

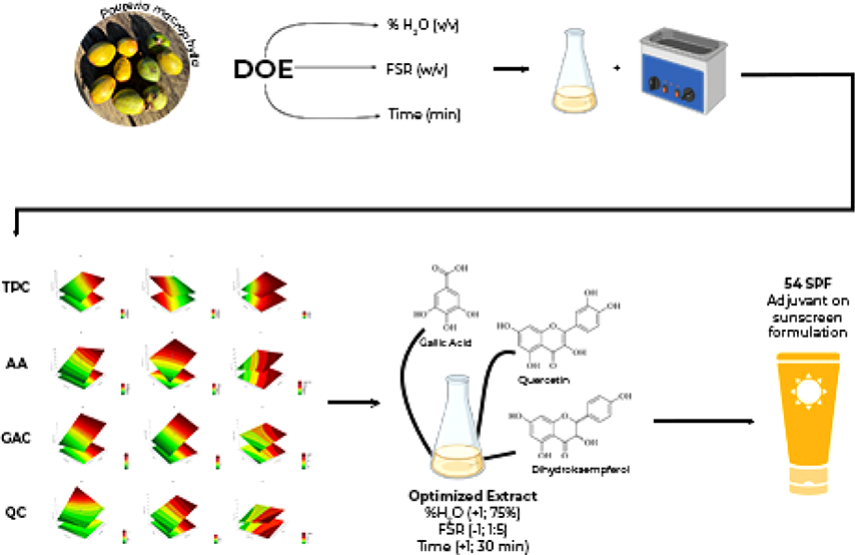

*Pouteria macrophylla*, also known as cutite, is an Amazonian fruit distributed in the western regions of North Brazil. Its fruits are rich in phenolic compounds, such as gallic acid (GA) and quercetin (Q), making it an excellent ingredient for cosmetic applications due to its high antioxidant activity and stability. A study optimized the extraction of GA and Q using hydroalcoholic ultrasound-assisted extracts by a central composite design, focusing on three independent variables: water–ethanol percentage (%H_2_O; v/v), fruit-solvent ratio (FSR; w/v), and time (*t*; min). Response surface methodology was used to identify the optimal conditions for maximizing gallic acid and quercetin content. Results showed antioxidant activity ranged from 1365.15 to 265.50 mg TE/mL and total phenolic compounds from 4293.7 to 897.04 mg GAE/L. A direct correlation between %H_2_O and FSR in the quercetin content response was observed. On the other hand, there was an inverse correlation between the FSR and the extraction of gallic acid, with a significance level of 90% (*p* < 0.1). The optimization of cutite hydroalcoholic extracts resulted in 10.22 ± 0.6 mg/L and 0.75 ± 0.25 mg/L for gallic acid and quercetin, respectively. Moreover, the optimized extract displayed a sun protection factor of 54, indicating its potential in cosmetic formulations and sunscreen products.

## 1 Introduction

Oxygen is essential for aerobic processes and acts as the primary electron acceptor. It is involved in the formation of various free radicals, such as reactive oxygen species (ROS). While moderate concentrations of free radicals are important for certain biological functions, an imbalance with antioxidant molecules can lead to oxidative stress, which is harmful to the body.^1,2^

Antioxidants are important molecules used to fight against the harmful effects of oxidative stress, which is a natural yet potentially harmful threat to our bodies. They can be sourced from within the body (endogenous) or from external sources (exogenous) and play a crucial role in reducing oxidative stress, preventing genetic mutations, and protecting against various forms of cellular damage.^3^ In the cosmetic industry, antioxidants are used both as preservatives to prolong product shelf life and as active ingredients to combat aging and skin issues. Plant extracts are gaining attention as natural alternatives to traditional antioxidants, containing beneficial bioactives like phenolic acids and flavonoids, although stability and yield challenges remain as researchers seek to optimize their use.^1,4^

The Amazonian ecosystem is renowned for its remarkable biodiversity. The fruits found in this ecosystem are considered functional foods that play significant roles in nutrition and protection. These fruits contain a high number of chemical compounds, such as polyphenols, which have strong antioxidant properties. These antioxidants help in preventing cellular disorders by slowing oxidation reactions. Oxidative stress can lead to cell death and genetic misregulation.^5^

*Pouteria macrophylla* (Lam.) Eyma, also known as cutite, is a plant species that grows naturally on the Andean slopes and in the western Amazon region. It belongs to the Sapotaceae family, and its fruits have a bright yellow pulp that measures around 2.5 to 3.5 cm in length. The fruits have an ellipsoid shape and a smooth, glabrous texture. The pulp fruit extracts of cutite are rich in gallic acid, quercetin, and other phenolic acids. These compounds have antioxidant properties and can inhibit free radicals, tyrosinase activity, and melanogenesis genes. As a result, cutite is an excellent ingredient for depigmentation in cosmetic formulations. Additionally, aqueous cutite extracts are highly stable, making them suitable for use in cosmetics.^6,7^

Gallic acid (GA) and quercetin (Q) are phenolic compounds identified in the cutite pulp extract. These compounds possess antioxidant properties and can be great reactive oxygen species (ROS) scavengers. Several studies have reported that gallic acid can inhibit melanin synthesis in melanoma cells and has the advantage of being a nonallergenic molecule.^8^ On the other hand, quercetin is a flavonol used to prevent and treat cancer, kidney and liver failure, and heart diseases. Its potent antioxidant activity can be attributed to the abundance of hydroxyl groups, which readily donate hydrogen atoms, and its capacity to chelate free metal ions. High concentrations of different quercetin derivatives, such as complexes with fatty acids like linoleic and linolenic acid, isoquercitrin, and hyperin, also lead to tyrosinase inhibition. Consequently, they can treat hyperpigmented skin and other melanin-related disorders.^9^

Extensive studies on the antioxidant capacity of quercetin and gallic acid make the use of extracts derived from natural products from the Amazon very compelling, especially when they are optimized and employed in the cosmetic industries, contributing to the Green Beauty Movement, which is increasing as the years pass. Society is changing its mindset toward sustainability, creating more concerned consumers who care not just about what they are using but also about the processes and how they deal with waste, so the cosmetic industry must follow the expectations that these consumers are creating.^10^ Due to the conflict between sustainability and the performance of cosmetic ingredients, this study aimed to optimize the extraction conditions of cutite fruits to improve the content of gallic acid and quercetin, two promising and natural actives for the cosmetic industry.

## 2 Materials and Methods

### 2.1 *Pouteria macrophylla* Fruits

*Pouteria macrophylla* (Lam.) Eyma fruits were collected in the metropolitan region of Belém (PA, Brazil) in March 2019. The botanical identification was performed by comparison with authentic *P. macrophylla* vouchers of the Museum Emílio Goeldi Herbarium (MG239766), and the species was registered in the Sistema Nacional de Gestão do Patrimônio Genético e do Conhecimento Tradicional Associado (SISGEN) under registration number AE3EA55. The harvested fruits were immediately washed with distilled water and sodium chloride solution (10%; v/v), ground, and stored in a freezer at −20 °C. The frozen fruits were freeze-dried for 48 h and stored at −20 °C until use.

### 2.2 Ultrasound-Assisted Extraction (UAE) of Gallic Acid and Quercetin

The optimal conditions for the UAE of gallic acid and quercetin from the freeze-dried *P. macrophylla* fruits were determined by response surface methodology (RSM) using a 2^3^ central composite design (CCD). Three independent variables were tested at two levels (−1 and +1): percentage of water in ethanol (25 to 75%, v/v) (%H_2_O; *x*_1_), fruit-solvent ratio (1:15 to 1:15, w/v) (FSR; *x*_2_), and time in the ultrasound bath (5 to 15 min) (t; *x*_3_). The CCD consisted of a 2^3^ factorial design plus three repetitions at the central point, totaling 11 runs (Table 1).

**Table 1:** Function of the Normalized Product Used in SPF Estimation (Sayre et al., 1979)

Wavelength (λ, nm)	EE × *I* (normalized)	Wavelength (λ, nm)	EE × *I* (normalized)
290	0.0150	310	0.1864
295	0.0817	315	0.0839
300	0.2874	320	0.0180
305	0.3278	total	1

For all the UAE experiments, an ultrasonic bath (7Lab, model SSBu 3,8L, São Paulo, Brazil) at room temperature (≈25 °C), with a fixed ultrasonic frequency of 40 kHz and 100 W of power, was used. An ethanol/water solution (v/v) was added to freeze-dried cutite fruits (0.5 g) at the chosen fruit-solvent ratio and directed to the ultrasonic bath during different residence times, according to the previously described parameters (Table 1), and the experimental responses were executed with the liquid extract. After the UAE procedure and statistical validation, the liquid portion of the optimized extract was separated from the solid residues and subjected to gallic acid and quercetin quantification, aiming to validate the optimal conditions, which were the main responses (dependent variables). In addition, the total phenolic compound content and antioxidant capacity by DPPH radical assay were also monitored.

#### 2.2.1 Gallic Acid and Quercetin Contents

Gallic acid and quercetin contents were determined by UV–visible spectroscopy at 260 nm in a spectrophotometer (Ultrospec 5300 pro – Amersham Bioscience). Standard curves were prepared by the solubilization of gallic acid in water (10.0, 7.5, 5.0, 2.5, and 1.0 μg/mL) and quercetin in ethanol (15.0, 10.0, 7.5, 5.0, and 1.0 μg/mL). The gallic acid and quercetin concentrations were obtained by the curve equation obtained by linear regression (*Y*_AG_ = 0.0786*x*; *R*^2^ = 0.996 and *Y*_Q_ = 0.0658*x*; *R*^2^ = 0.999).

#### 2.2.2 Total Phenolic Compound (TPC) Content

The amount of total phenolic compounds in hydroalcoholic cutite extracts was determined using the Folin–Ciocalteu method.^11,12^ The extracts were dissolved at 1 mg/mL and diluted with water. To each sample (500 μL), 250 μL of Folin–Ciocalteu (1.0 N) and 1250 μL of sodium carbonate (Na_2_CO_3_) (0.075 mg/mL) were added. The mixture was left undisturbed in a dark room for 30 min. After that, the absorbance was measured at 760 nm and 25 °C (Ultrospec 5300 pro – Amersham Bioscience). Gallic acid was used to prepare the experimental calibration curve at concentrations of 0.0, 2.0, 4.0, 8.0, 16.0, 24.0, 32.0, and 40.0 mg/mL under the same conditions. The total phenolic content was expressed in milligrams per gram of sample (mg GAE/g) and calculated as gallic acid equivalents (GAE).

#### 2.2.3 DPPH Radical Scavenging

The antioxidant activity of the ethanolic cutite extracts was evaluated by the DPPH radical scavenging method.^13^ The extracts were solubilized in methanol, and aliquots of 50 μL were mixed with 1950 μL of DPPH solution (60 μM). The absorbance was measured after 30 min at 517 nm, and the DPPH radical scavenging inhibition was calculated in relation to the negative control. After the inhibition percentage values of the extract were obtained, they were further transformed into Trolox equivalents per milliliter of extract (TE/mL) using an analytical standard curve. This standard curve was developed within the 1 to 40 mM concentration range.

### 2.3 Tyrosinase Inhibition Assay

The inhibition of the tyrosinase enzyme was determined by the dopachrome method using l-tyrosine as the substrate.^14^ In a microplate, 20 μL of sample (1, 0.5, 0.25, 0.125, and 0.06 mg/mL) and tyrosinase solution (0.1 mg/mL) were added to 40 μL of substrate and 80 μL of phosphate buffer (pH 6.8). The reaction mixture was incubated for 30 min at 37 °C. After the reaction time, the absorbance was read at 492 nm, and the inhibition percentage was calculated in relation to the control. Phosphate buffer and kojic acid were tested under the same conditions as the negative and positive controls, respectively.

### 2.4 Sun Protection Factor Determination

Sun protection factor (SPF) was determined in vitro and quantified by UV–visible spectrophotometry.^15^ A solution of the optimized extract was prepared in water at 1 mg/mL to perform the determination of SPF, which was measured by analyzing the absorption spectrum of UVB rays in a wavelength range between 290 and 320 nm. The SPF was estimated by a mathematical equation (eq 1; Table 1).
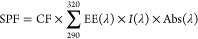
1

Where the correction factor is a constant represented by the abbreviation CF and has a fixed value of 10 and the erythematogenous effect (EE) of sun radiation in each wavelength is a product of the intensity (*I*) of sun radiation at each wavelength along with the absorbance reading (Abs) from the sample in each wavelength (λ). The sum of this product, multiplied by the CF constant, will result in the protection factor of the analyzed sample.

### 2.5 Chemical Characterization of the Optimized Extract by Liquid Chromatography—Mass Spectrometry (LC-ESI-MS)

Chemical characterization was performed by LC-ESI-MS with a spectrometer (Bruker, Massachusetts, USA). The chromatographic analysis was performed on a Luna 5 μm C18 100 Å column (250 × 4.6 mm, Phenomenex, Torrance, USA). The binary gradient mobile phase consisted of 0.1% formic acid (Sigma-Aldrich, St. Louis, MO, USA) in water (solvent A) and 0.1% formic acid in methanol (Sigma-Aldrich, St. Louis, MO, USA) (solvent B). Compounds were eluted from the analytical column with a 50 min gradient ranging from 5% to 100% solvent B at a constant 1 mL/min flow rate. The column compartment temperature was set to 40 °C. Data acquisition was performed in positive and negative ionization mode, with fragmentation in multiple stages (MS^2^ and MS^3^), according to the following parameters: nebulization gas pressure, 50.0 psi; capillary temperature, 300 °C; transfer capillary input voltage, −4500 V; desolvation gas, nitrogen (N_2_), flow 10 L/min; collision gas, helium (He); range acquisition, *m*/*z* 50–1200. Raw data were analyzed using Data Analysis 4.3 (Bruker, Massachusetts, USA).

### 2.6 Statistical Analysis

The results of the experimental design were analyzed using the Statistica 7.0 software (Statsoft Inc., USA), and all experimental data obtained by the CCD were fitted to the second-order polynomial model (eq 6):
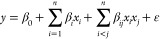
2where “*y*” is the dependent variable and β is the coefficient of the regression model of each term. The adequacy of the second-order model was determined by evaluating the coefficient of determination (*R*^2^), lack of fit, and Fisher test values (*F*-value) through Analysis of Variance (ANOVA) at 10% statistical significance (α = 0.1).

## 3 Results and Discussion

### 3.1 Fitting the Model

In this experiment, 11 different tests were conducted to analyze and optimize the variations in the independent variables, which led to different results for the dependent variables. The dependent variables included total phenolic content (303.91–4293.70 mg GAE/L), antioxidant activity (265.50–1406.72 mg TE/mL), gallic acid (2.835–9.137 mg GA/L), and quercetin content (0.319–1.464 mg Q/L). The results for each of these variables are shown in Table 2. The data collected from the Central Composite Design (2^3^) were fitted to quadratic models and used to interpret the results.

**Table 2:** Values of Dependent Variables Obtained from the Variation of Independent Variables^a^

Central composite design (CCD) **-***Pouteria macrophylla*							
	Independent variables			Dependent variables			
Run	% H_2_O(v/v)	FSR (w/v)	*t* (min)	TPC (mg GAE/L)	AA (mg TE/mL)	GAC (mg AG/L)	QC (mg Q/L)
1	25 (−1)	5 (−1)	10 (−1)	2146.85	1406.72	9.137	0.740
2	75 (+1)	5 (−1)	10 (−1)	1119.41	1167.10	8.477	1.135
3	25 (−1)	15 (+1)	10 (−1)	897.04	768.56	4.787	0.484
4	75 (+1)	15 (+1)	10 (−1)	303.91	744.11	2.853	0.382
5	25 (−1)	5 (−1)	30 (+1)	2614.93	1365.15	8.524	0.681
6	75 (+1)	5 (−1)	30 (+1)	2280.00	967.43	8.406	1.464
7	25 (−1)	15 (+1)	30 (+1)	1026.53	326.01	4.445	0.392
8	75 (+1)	15 (+1)	30 (+1)	1081.66	265.50	3.561	0.319
9 (CP)	50 (0)	10 (0)	20 (0)	3338.33	1079.08	6.107	0.776
10 (CP)	50 (0)	10 (0)	20 (0)	4159.18	1119.83	5.754	0.737
11 (CP)	50 (0)	10 (0)	20 (0)	4293.70	933.19	5.058	0.707

a %H_2_O = ethanol/water solution; FSR = fruit-solvent ratio; *t* = time; TPCs = total phenolic compounds; AA = antioxidant activity; GAC = gallic acid content; QC = quercetin content; CP = central point.

The statistical parameters of total phenolic content (R^2^ = 0.971), antioxidant activity (*R*^2^ = 0.986), gallic acid, and quercetin content (*R*^2^ = 0.969 and *R*^2^ = 0.981, respectively) were determined using analysis of variance (ANOVA) and *F*-test (Table S1). The responses generated significant and predictive polynomial models, which allowed for the assessment of the quality and suitability of each independent variable. Table S2 displays the regression coefficients and their corresponding *p*-values, considered significant when less than 0.1. These values indicate the effect of each independent variable on the responses.

### 3.2 Effect of the Variables in the UAE of Phenolic Compounds

A polynomial model for TPC response was constructed using multiple regressions, and eq 3 shows the relationship between the effects and TPC response. Phenolic compounds are widespread in nature and can be found in fruits, leaves, and many other plant-based sources. Several studies have linked their chemical structures to antioxidant activity.^16^

The ultrasound-assisted extraction technique with a mixture of ethanol and water as the solvent resulted in high total phenolic content (TPC) values in experiment 11 (4293.70 mg GAE/L), located at the central point of the experimental design. The optimal conditions for a high concentration of phenolic compounds were achieved with 50% water in ethanol, 1:10 FSR (w/v), and 20 min in the ultrasound bath. Ethanol extracts of *Pouteria* fruit have been reported to contain phenolic compounds. *Pouteria caimito* and *P. campechiana*, collected in Roraima (Brazil) and Bandarban district (Bangladesh), exhibited 134.4 ± 9.1 mg GAE/100 g^17^ and TPC 205 mg GAE/100 g, respectively. Although these values are significant, they are lower than those obtained in experiment 11.^18^


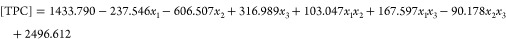
3

The only significant factor affecting the TPC response was the fruit-solvent ratio, shown in Figures S1 and 1 (−606.507; *p* = 0.080111). The Pareto charts demonstrate an inverse correlation between the TPC and FSR values, meaning that when the FSR values are lower, the TPC values are higher. This behavior is also observed in experiments 1, 2, 5, and 6, which displayed high values for phenolic content. Although it is unpredictable to anticipate the behavior of natural bioproducts in a solvent-material extraction system, due to the chemical composition of solvents and the diversity of compounds and their structures, higher content of phenolic compounds was observed when the FSR was lower in almond hull (*Prunus amygdalus*) extracts and grape byproducts.^19^

**Figure 1:**
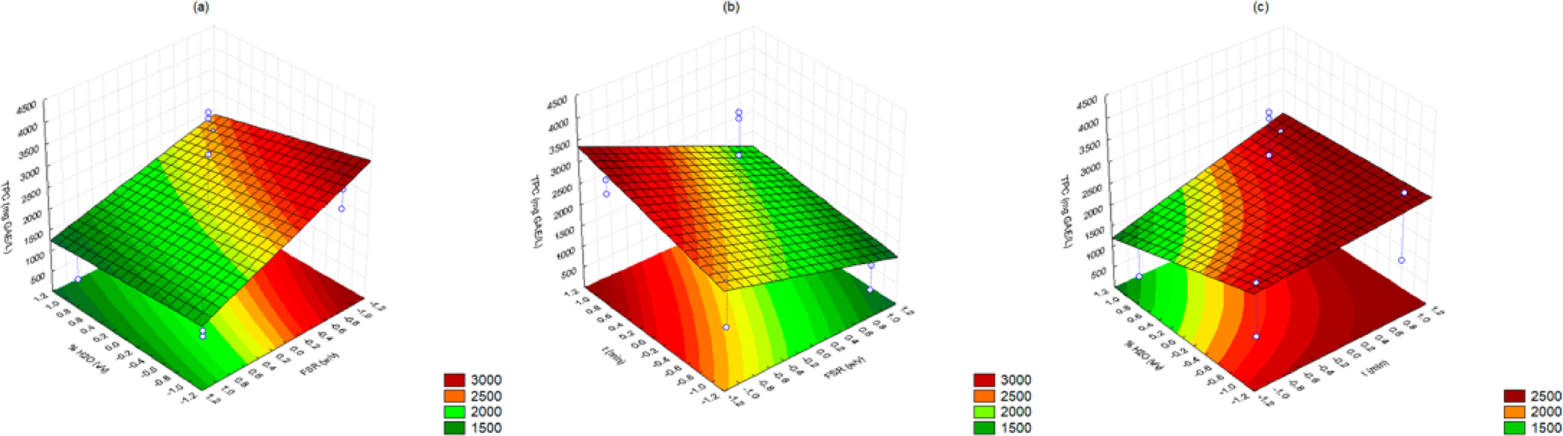
Response surface methodology of total phenolic compound response charts relating to (a) FSR and %H_2_O, (b) time and FSR, and (c) %H_2_O and time.

### 3.3 Effect of the Variables on Antioxidant Activity

The experiment conducted showed significant findings in antioxidant activity. Specifically, in experiment 1, when 25% water in ethanol solution (v/v), an FSR of 1:5 (w/v), and 10 min of ultrasound bath were applied, it exhibited 1406.72 mg TE/mL scavenging of DPPH radicals. It is worth noting that the *Pouteria* genus is widely known for its excellent antioxidant activity. Previous studies have already described the *P. glomerata* from Mato Grosso do Sul, Brazil hydroalcoholic extracts with 30 707 ± 1774 mmol TE/100 g and 133.25 μg TE/g for *P. macrophylla* from Pará, Brazil ethanolic extract.^7,20^

The presence of phenolic compounds in the nucleus acts as an efficient sensor of reactive species, creating a strong relationship between antioxidant activity and phenolic content^16^ as can be seen in this experimental design (Table 2), mainly in experiments 1, 2, 5, 9, 10, and 11, which exhibit a preference for the solubilization of these compounds in alcohol and aqueous mixtures. Ethanol has an intermediate polarity, which permits the extraction of both phenolic compounds, lipophilic and hydrophilic ones. Additionally, this solvent enhances the permeability of cell matrices and increases the diffusion of compounds through solid–liquid extraction.^21^
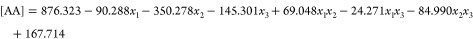
4

The polynomial equation of AA (eq 4), generated using regression coefficients, has revealed an inverse correlation between the FSR and time effects and the antioxidant capacity of cutite extracts, as seen in experiment 1. By analyzing the Pareto and surface response charts (Figures S1 and 2, respectively), it is possible to observe that AA exhibited significant and inverse regression coefficient values for the FSR (−2146.85; *p* = 0.0097) and time (−145.301; *p* = 0.0523). These results indicate that as these effects decrease, the antioxidant activity of the tested sample increases.

**Figure 2:**
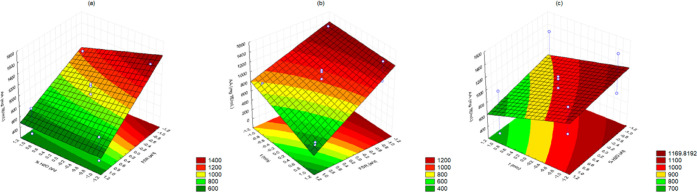
Response surface methodology of antioxidant activity response charts relating to (a) FSR and %H_2_O, (b) time and FSR, and (c) %H_2_O and time.

UAE is reported to be more effective than conventional extraction methods because of the cavitation phenomenon, where the amplitude of the ultrasound frequency ruptures the cell walls. This process enhances the bioactive compounds extracted from the fruit matrices.^21^ Although it is an ideal method of bioactive extraction, the loss of antioxidant activity in this study, due to the increase of FSR and time (min) in the ultrasound bath, may be due to the degradation of bioactive compounds by the amplitude of ultrasound frequency and extraction time.^22^

### 3.4 Effects of the Variables in UAE of Gallic Acid and Quercetin

The literature has already described the presence of gallic acid as a primary compound in cutite extracts. In this study, the highest amount of gallic acid obtained from hydroalcoholic solutions was around 9.137 mg of GA/L. However, an aqueous ultrasound-assisted extract contained 12.47 mg of GA/g from the fruit dry matter. Other fruits from the *Pouteria* genus also contain gallic acid in their chemical composition. For instance, *P. sapota* has an amount of 0.17 mg GA/g, much lower than the content found in the cutite fruit extract.^23^

As it is known, gallic acid is a phenolic compound with phenol and carboxylic acid properties; it is an organic acid with three adjacent hydroxyl groups and a carboxyl group.^24^ The relationship between the GAC response and FSR was examined, and it was found that the FSR had a significant inverse influence on GAC response (−0.423; *p* = 0.065). The regression coefficient for these effects indicated that the GAC response increased as the FSR values decreased. These results suggest that low FSR values significantly impact the GAC response more than high FSR values.

**Figure 3:**
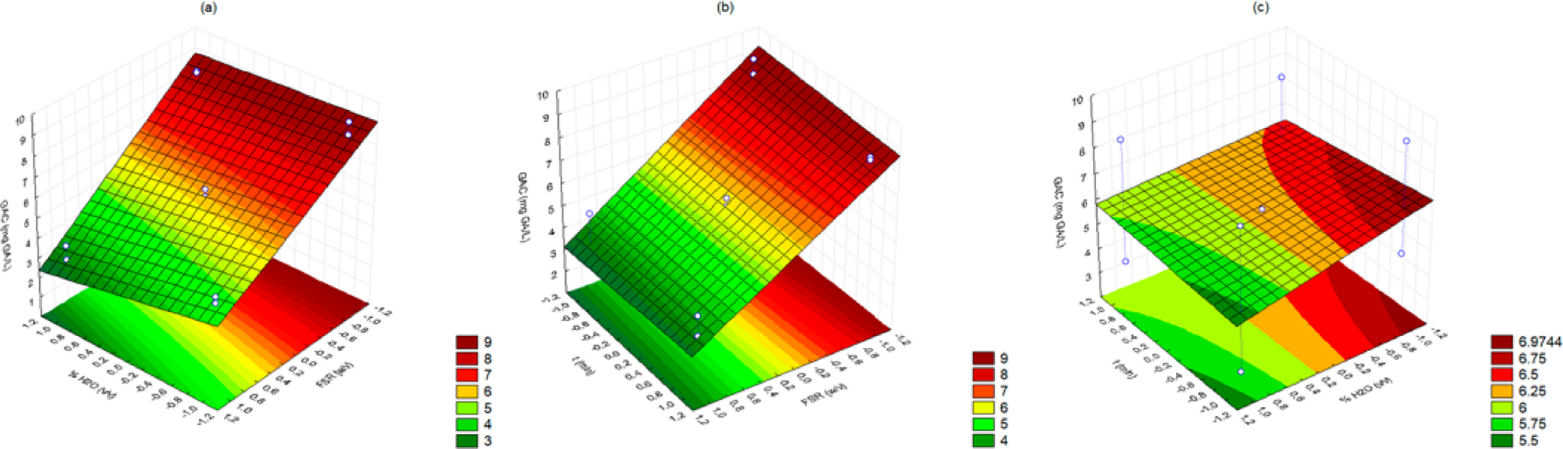
Response surface methodology of gallic acid content response charts relating to (a) FSR and %H_2_O, (b) time and FSR, and (c) %H_2_O and time.

Therefore, to improve the GAC response, it is essential to maintain a lower FSR value (1:5; w/v). The GAC response had only the FSR as a significant influence (−0.423; *p* = 0.065). The regression coefficient for these effects indicated that this correlation is inversely proportional; thus, when the FSR values are lower, there is a higher GAC response (Figure 3). Equation 5 indicates the relationship between effects and responses.
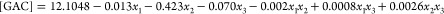
5

**Figure 4:**
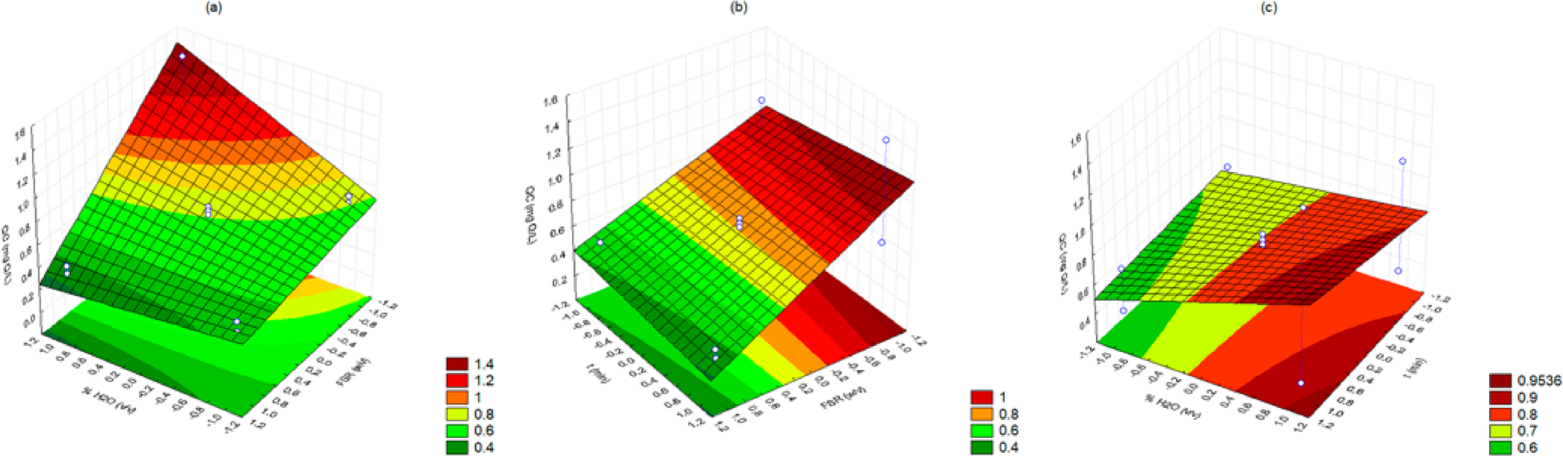
Response surface methodology of quercetin content response charts relating to (a) FSR and %H_2_O, (b) time and FSR, and (c) %H_2_O and time.

Quercetin is a flavonol type with three aromatic rings and three hydroxyl groups connected to its backbone. This compound possesses high antioxidative properties, as it can help eliminate reactive oxygen species.^25^Figure 4 shows the results of the dependent variable related to QC, which indicate that only the independent variable “time” was not significantly influential (*p* = 0.705). On the other hand, there were significant regression values for %H_2_O (0.014; *p* = 0.0103) and SLR (0.028; *p* = 0.0637), with a direct and inverse proportion, respectively. Therefore, higher values of QC were obtained when the solution of ethanol/water had a higher concentration of water and a lower solid–liquid ratio.
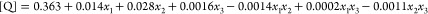
6

QC responses suggested different results, where only time (*p* = 0.705) did not have a significant influence. However, the regression values for %H_2_O (0.014; *p* = 0.0103) and FSR (0.028; *p* = 0.0637) displayed a direct and inverse influence, respectively. For this, higher values of QC were obtained when the solution of ethanol/water had higher concentrations of water and a lower ratio of fruit-solvent, represented in Table 2 by experiment 6, when the quercetin content in the sample reached 1.464 mg/L.

A previous study described that higher FSR values result in a greater concentration gradient during solid–liquid extraction procedures, accelerating the diffusion of solutes from the material into the liquid phase. It was also described that high ethanol concentrations in water solutions might prioritize extracting more polar phenolic compounds by increasing polarity, leading to better solubilization, corroborating our results.^26^

### 3.5 Best Conditions for Simultaneous Extraction of Gallic Acid and Quercetin

The desirability function was used to maximize the dependent variables obtained from fixation of the parameters proposed by the other responses. A rate from 0 to 1 can be obtained from this function, where values close to 0 indicate an unacceptable desirability, and values close to 1 suggest high desirability. Once the desirability (*d*) of each response is found, the global desirability (*D*) is obtained from the mean square of individual desirability, maximizing several responses.^27^ The obtained statistics showed a value of 0.92 regarding global desirability, which indicates a good fit for optimization adjustment, showing that the operational conditions are the most adequate inside the experimental domain.

**Figure 5:**
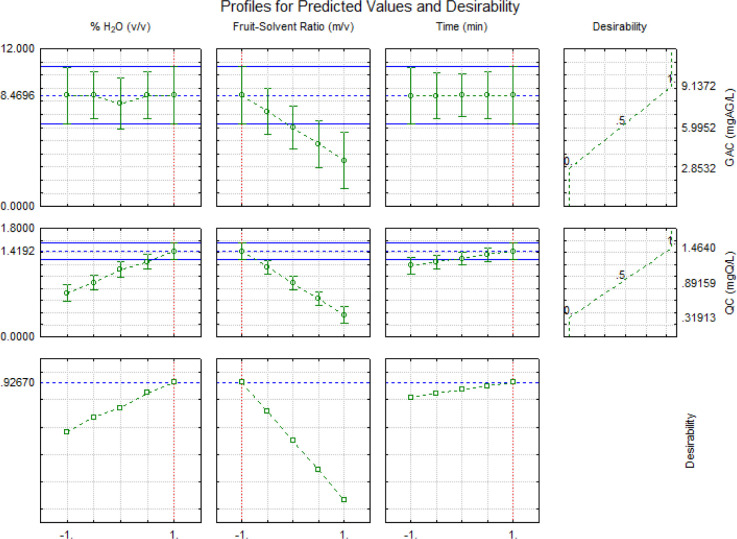
Values of desirability function for gallic acid and quercetin content. %H_2_O = ethanol/water solution; FSR= fruit-solvent ratio; *t* = time; “–1” (lower value of independent variables) = 25%, 1:5 and 10 min; and “1” (highest value of independent variables).

In this experiment, the means of GAC and QC were within the predicted confidence range (90%) for the desirability function. The best condition for extracting gallic acid and quercetin from cutite was 75% water in ethanol, with a solid–liquid ratio of 1:5 (w/v) for 30 min using ultrasound assistance. An experiment was performed in triplicate with the same conditions as indicated in Figure 5 to verify these optimal conditions. The experimental values found were 10.22 ± 0.6 mg AG/L and 0.74 ± 0.25 mg Q/L for GAC and QC, respectively. The relative error for the GAC response was low (6.9%), but for the QC response, it was considerable (23.3%).

### 3.6 Identification of Compounds Present in the *Pouteria macrophylla* Extract by LC-ESI-IT/MS

18 well-defined peaks and 12 different compounds were identified in the optimized *P. macrophylla* ethanolic extract by liquid chromatography with mass spectrometry, compared with the literature. The LC-MS chromatogram (Figure S2) and Table 3 show the identification data.

**Table 3:** Compounds Identified in the *Pouteria macrophylla* Hydroalcoholic Extract by HPLC-ESI-MS

Peak	Time retention (min)	Compound	Ionization mode	Fragments	References
1	2.1	trisaccharide	+	527 [M + Na]^+^; 509; 365;^a^ 347; 203; 185	(32)
2	2.3	caffeoyl-*O*-hexoside	–	683 [2M – H]^−^; 341^a^ [M – H]^−^; 221; 179; 161	(33)
			+	707 [2M + Na]^+^; 365^a^; 203; 185	
3	2.7	galloyl-*O*-hexoside	–	331 [M – H]^−^; 211; 169; 125;^a^ 107	(28)
4	2.8	galloyl quinic acid	–	343 [M – H]^−^; 191;^a^ 169; 125; 107	(29)
5	3.2	gallic acid	–	339 [2M – H]^−^; 169 [M – H]^−^; 125^a^	(23)
6	6.0	HHDP-hexoside	–	481 [M – H]^−^; 355; 329; 301;^a^ 283; 273; 255; 215	(30,34)
			+	483 [M + H]^+^; 321; 303; 275;^a^ 247; 229; 195	
7	6.7	HHDP-hexoside isomer I	–	481 [M – H]^−^; 355; 329; 301;^a^ 283; 273; 255; 215; 151	
8	8.7	galloyl-HHDP-hexoside	–	633 [M – H]^−^; 481; 301; 271;^a^ 215	(30)
9	9.3	HHDP-hexoside isomer II	–	481 [M – H]^−^; 329; 301;^a^ 283; 273; 255; 215; 151	(30,34)
10	9.6	taxifolin-*O*-hexoside	–	465 [M – H]^−^; 285;^a^ 241; 151	(35)
11	10.3	taxifolin-*O*-hexoside isomer I	–	465 [M – H]^−^; 285;^a^ 241; 151	(35)
12	11.2	galloyl-HHDP-hexoside isomer	–	633 [M – H]^−^; 481; 331; 301;^a^ 271; 215	(30)
13	11.9	taxifolin-*O*-hexoside isomer II	–	465 [M – H]^−^; 285;^a^ 241; 151	(35)
14	12.6	dihydrokaempferol-*O*-hexoside	–	449 [M – H]^−^; 269;^a^ 241	(35)
15	13.5	myricetin-*O*-hexoside	–	479 [M – H]^−^; 316;^a^ 287; 271	(36)
16	13.9	dihydrokaempferol-*O*-hexoside	–	449 [M – H]^−^; 269;^a^ 241	(35)
17	16.8	dihydrokaempferol	–	575 [2M – H]^−^; 287;^a^ 269; 259; 243; 125	(35)
18	22.0	quercetin	–	301 [M – H]^−^; 255; 227; 149;^a^ 107	(37)
			+	303^a^ [M + H]^+^; 285; 257; 229; 203; 177; 153	

a Ion with 100% abundance.

In the optimized extract of *P. macrophylla*, a total of 18 compounds identified by liquid chromatography with mass spectrometry were compared with the literature. Predominantly, the compounds found are gallic acid derivatives, characterized as esters of gallic acid and polyol, commonly glycosylated. Additionally, some flavonols were identified. This study identified some gallotannins, such as galloyl-*O*-hexoside, HHDP-hexoside, and their respective derivatives. These compounds showed the characteristic fragment ions in their product ion spectra by consecutive elimination of the gallate unit.

Compound 5 produced an [M – H]^−^ ion at *m*/*z* 169 and 125, characteristic of gallic acid, which was confirmed with an authentic standard in the quantification. Compound 5 produced the most prominent and well-defined peak, corroborating with UV–vis spectrophotometry quantification displayed in Table 3, used to quantify the gallic acid content in this optimized hydroalcoholic extract. It presented itself as the most abundant compound in the extract. Besides this, it was possible to note its derivatives (galloyl-*O*-hexoside, galloyl quinic acid, galloyl-HHDP-hexoside) also in the optimized extract, described in the literature with similar retention times and fragments.^28−30^

Identifying some flavonols in the optimized hydroalcoholic extract of *P. macrophylla* was also possible. Peak 18, with a retention time of 22 min, produced [M – H]^−^ in *m*/*z* 301, which was found to contain quercetin tentatively.^31^ Although it was a small peak, it was well-defined and corroborated with UV spectrophotometry, which showed small amounts of quercetin in the optimized extract (Table 1).

### 3.7 Tyrosinase Inhibition

The inhibition of the tyrosinase enzyme was evaluated in experiment 6 using l-tyrosine as the substrate and kojic acid as the positive control – a well-established inhibitor. The extract displayed an IC_50_ value of 495.1 μg/mL for the analyzed extract, with a range of inhibition varying from 11.8 to 49.03%, indicating weak inhibition.

The ethanolic extracts from the leaves of *P. torta* and ethyl acetate extracts of *P. campechiana* showed IC_50_ values of 258.53 and 828.54 μg/mL, respectively.^38^ The levels of tyrosinase inhibition can be influenced by the polarity of solvents applied to extraction. Methanolic extracts exhibited higher inhibitory activity against tyrosinase.^39^ Some studies suggest that compounds with hydroxyl groups in the “para” position effectively inhibited tyrosinase activity, a common pattern in many types of polyphenols. Conversely, glycosylated polyphenols tend to lack inhibitory activity.^40^ The moderate polarity of the extract obtained from a 1:3 mixture of EtOH:H_2_O produced a complex mixture of compounds, including phenolics with strong antityrosinase activity and glycosylated compounds that potentially reduced the overall inhibitory effect.

### 3.8 Sun Protection Factor Determination

Sun protection factor (SPF) measures the effectiveness of sunscreen products. According to the Brazilian Health Regulatory Agency (ANVISA; RDC n° 30/2012), SPF can be classified as low protection (6 to 14.9), medium protection (15 to 29.9), high protection (30 to 50 SPF), and extra high protection (higher than 50 and lower than 100 SPF). Although ANVISA does not recommend a specific range of SPF, the US Food and Drug Administration (FDA) recommends using sunscreen formulations with an SPF of at least 30 to 50 due to the underapplication of sunscreens in real life.^41^

From the absorbances obtained by spectrophotometric analyses, the sun protection factor found was 54, which matched the extra high protection. Ethanolic extracts of cutite showed high levels of phenolic compounds in their chemical composition. As it is known, these compounds can absorb UV radiation as well as their derivatives.^42^ A previous study described that the stilbenes (retinol and piceid), flavonols (catechin, quercetin, kaempferol, galangin, apigenin, naringenin, chrysin, and pinocembrin), and hydroxycinnamic acids (coumaric acid, ferulic acid, caffeic acid, caffeic acid phenyl ester, dimethyl caffeic acid) in a concentration of 10 mM showed absorption of UV rays at a rate of 7 to 29, corresponding to low and medium sun protection.^43^

In the optimized cutite ethanolic extract, high concentrations of gallic acid (8.406 mg/L) were observed, and it was previously clarified that epithelial cells irradiated with UVB rays and damaged by the high production of reactive oxygen species from this radiation were treated with 1 and 10 μM of gallic acid and presented a decrease of 10 and 45% in the injuries, respectively.^44^

Phenolic compounds in plants have chromophores in their chemical structures that can absorb UV radiation and visible light, leading to a biological response. The optimized cutite extract (1.464 mg Q/L) contains quercetin, which is capable of absorbing UVA radiation (λ = 365 nm) and UVC (λ = 256 nm). The chromophores directly absorb these rays, and the resulting energy is dissipated as light, heat, or as its decomposition into 2,4,6-trihydroxybenzaldehyde, 2-(3′,4′-dihydroxybenzoyloxy)-4,6-dihydroxybenzoic acid, and 3,4-dihydroxyphenylethanol. Topical application of quercetin has shown its antioxidant potential by preventing skin damage induced by UVB radiation and liposome peroxidation induced by UVC rays. The effects of a formulation that contains 10% quercetin and rutin have been compared to homosalate, an organic and synthetic filter used in the cosmetic industry.^45^

## 4 Conclusion

*Pouteria macrophylla* extracts are rich in bioactive compounds and have diverse applications. In this study, the extraction of gallic acid and quercetin from cutite hydroalcoholic extract was optimized, and it was found that the process can extract additional beneficial compounds, including dihydrokaempferol, glycerides of gallic acid, and other phenolic acids. The optimal conditions were: 75% water in ethanol (v/v) solution and a 1:5 (w/v) solid–liquid ratio for 30 min. The optimized extract exhibited a high sun protection factor and is a potent asset in the cosmetic industry. These compounds are also a great source of phenolic compounds and antioxidants, which prevent oxidative stress.

## Supporting Information Available

Pareto charts for standardized effect estimates (PDF)
UV–vis chromatogram of the *P. macrophylla* extract (PDF)
Pareto charts of the independent variables (Figure S1); UV–vis chromatogram of the *P. macrophylla* extract (Figure S2); ANOVA for the effects of ultrasound-assisted extraction (Table S1); regression coefficients for dependent variables of the *P. macrophylla* extract experiment design (Table S2) (PDF)
