# Supplementary figures and images for: Optimization of Extraction Conditions for Improving Gallic Acid and Quercetin Content in Pouteria macrophylla Fruits: A Promising Cosmetic Ingredient
Source: ACS Omega. 2025 Feb 13;10(7):7371–80. doi: 10.1021/acsomega.4c11241 (PMC11866184; doi:10.1021/acsomega.4c11241)

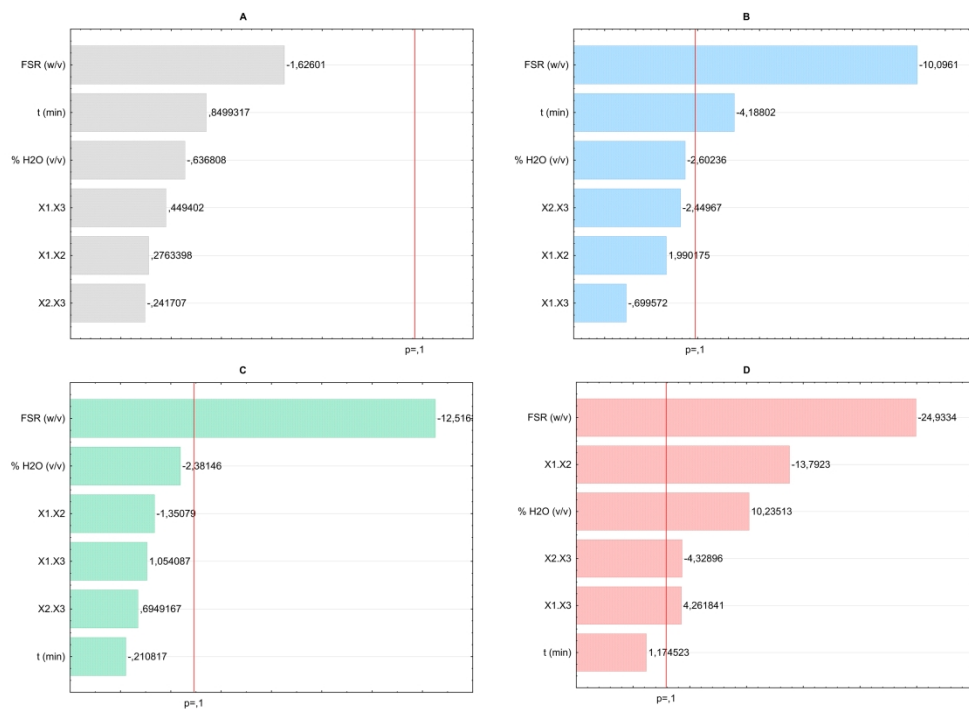

297x209mm (300 x 300 DPI)

Supplement: Supplementary file 1 — ao4c11241_si_001.pdf [file ao4c11241_si_001.pdf]

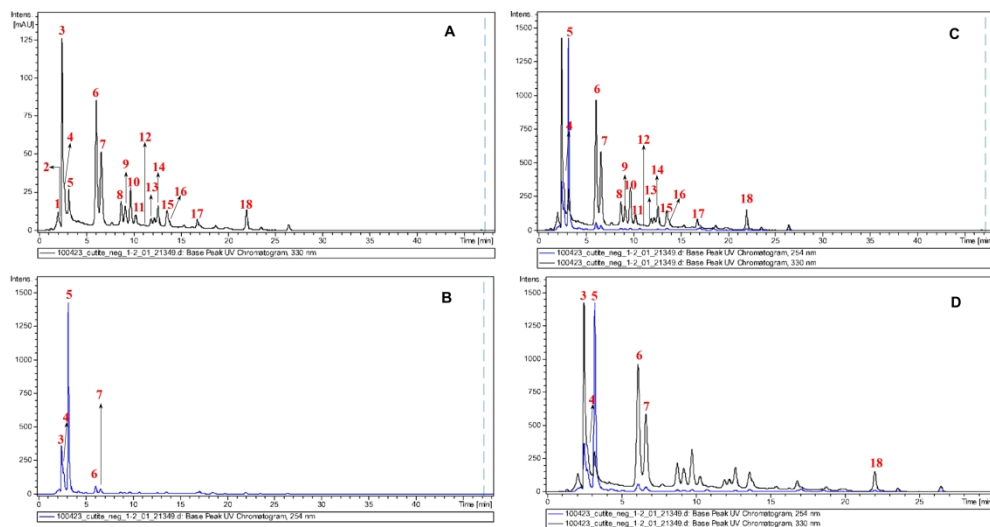

297x157mm (300 x 300 DPI)

Supplement: Supplementary file 2 — ao4c11241_si_002.pdf [file ao4c11241_si_002.pdf]
