# Supplementary material for: Optimization of Extraction Conditions for Improving Gallic Acid and Quercetin Content in Pouteria macrophylla Fruits: A Promising Cosmetic Ingredient
Source: ACS Omega. 2025 Feb 13;10(7):7371–80. doi: 10.1021/acsomega.4c11241 (PMC11866184; doi:10.1021/acsomega.4c11241)
Supplement: Supplementary file 3 — ao4c11241_si_003.pdf [file ao4c11241_si_003.pdf]

Supporting Information

Optimization of extraction conditions for improving gallic acid and quercetin content in *Pouteria macrophylla* fruits: a promising cosmetic ingredient

Camila F. B. Albuquerque[1], Dayenne A. A. de Souza[2], Pablo Luis B. Figueiredo[3], Cláudia Quintino Rocha[4], José Guilherme S. Maia[2], Massuo J. Kato[5], Renan Campos Chisté[6], Joyce Kelly R. da Silva[1,2]*

Camila Fernanda Barbosa Albuquerque

[1]Programa de Pós-Graduação em Biotecnologia, Instituto de Ciências Biológicas, Universidade Federal do Pará, Belém, PA 66075-110, Brazil.
E-mail: camila.barbosa@icb.ufpa.br
ORCID: https://orcid.org/0000-0003-1148-2968

Dayenne Alexsa Araújo de Souza

[2]Programa de Pós-Graduação em Química, Instituto de Química, Universidade Federal do Pará, Belém, PA 66075-110, Brazil.
E-mail: dayenne@ufpa.br
ORCID: https://orcid.org/0009-0002-2272-4133

Pablo Luis B. Figueiredo

[3]Laboratório de Química dos Produtos Naturais, Universidade do Estado do Pará, Belém, PA 66095-015, Brazil.
E-mail: pablo.figueiredo@uepa.br
ORCID: https://orcid.org/0000-0003-1365-3513

Claudia Quintino Rocha

[4]Programa de Pós-Graduação em Química, Universidade Federal do Maranhão, São Luís, MA 65085-580, Brazil.
E-mail: rocha.claudia@ufma.br
ORCID: https://orcid.org/0000-0002-3578-1869

José Guilherme S. Maia

[2]Programa de Pós-Graduação em Química, Instituto de Química, Universidade Federal do Pará, Belém, PA 66075-110, Brazil.
E-mail: gmaia@ufpa.br
ORCID: https://orcid.org/0000-0003-2201-8570

Massuo J. Kato

[5]Laboratório de Química de Produtos Naturais, Instituto de Química, Universidade de São Paulo, São Paulo, SP 05508-000, Brazil.
E-mail: majokato@iq.usp.br
ORCID: https://orcid.org/0000-0002-3315-2129

Renan Campos Chisté

[6]Faculdade de Farmácia, Universidade Federal de Minas Gerais, Belo Horizonte, MG 31270-901, Brazil.
E-mail: rchiste@ufmg.br
ORCID: https://orcid.org/0000-0002-4549-3297

Joyce Kelly R. da Silva

[1]Programa de Pós-Graduação em Biotecnologia, Instituto de Ciências Biológicas, Universidade Federal do Pará, Belém, PA 66075-110, Brazil.
[2]Programa de Pós-Graduação em Química, Instituto de Química, Universidade Federal do Pará, Belém, PA 66075-110, Brazil.
E-mail: joycekellys@ufpa.br
ORCID: https://orcid.org/0000-0002-9351-876X

Optimization of extraction conditions for improving gallic acid and quercetin content in *Pouteria macrophylla* fruits: a promising cosmetic ingredient

Camila F. B. Albuquerque[1], *Dayenne A. A. de Souza*[2], *Pablo Luis B. Figueiredo*[3], *Cláudia Quintino Rocha*[4], *José Guilherme S. Maia*[2], *Massuo J. Kato*[5], *Renan Campos Chisté*[1], *Joyce Kelly R. da Silva*[1,2*]

[1]*Programa de Pós-Graduação em Biotecnologia, Instituto de Ciências Biológicas, Universidade Federal do Pará, Belém, PA 66075-110, Brazil.*

[2]*Programa de Pós-Graduação em Química, Instituto de Química, Universidade Federal do Pará, Belém, PA 66075-110, Brazil.*

[3]*Programa de Pós-Graduação em Ciências Farmacêuticas, Instituto de Ciências da Saúde, Universidade Federal do Pará, Belém, PA 66075-110, Brazil.*

[4]*Programa de Pós-Graduação em Química, Universidade Federal do Maranhão, São Luís, MA 65085-580, Brazil.*

[5]*Laboratório de Química de Produtos Naturais, Instituto de Química, Universidade de São Paulo, São Paulo, SP 05508-000, Brazil.*

[6]*Faculdade de Farmácia, Universidade Federal de Minas Gerais, Belo Horizonte, MG 31270-901, Brazil.*

[*]*Corresponding Author: joycekellys@ufpa.br*

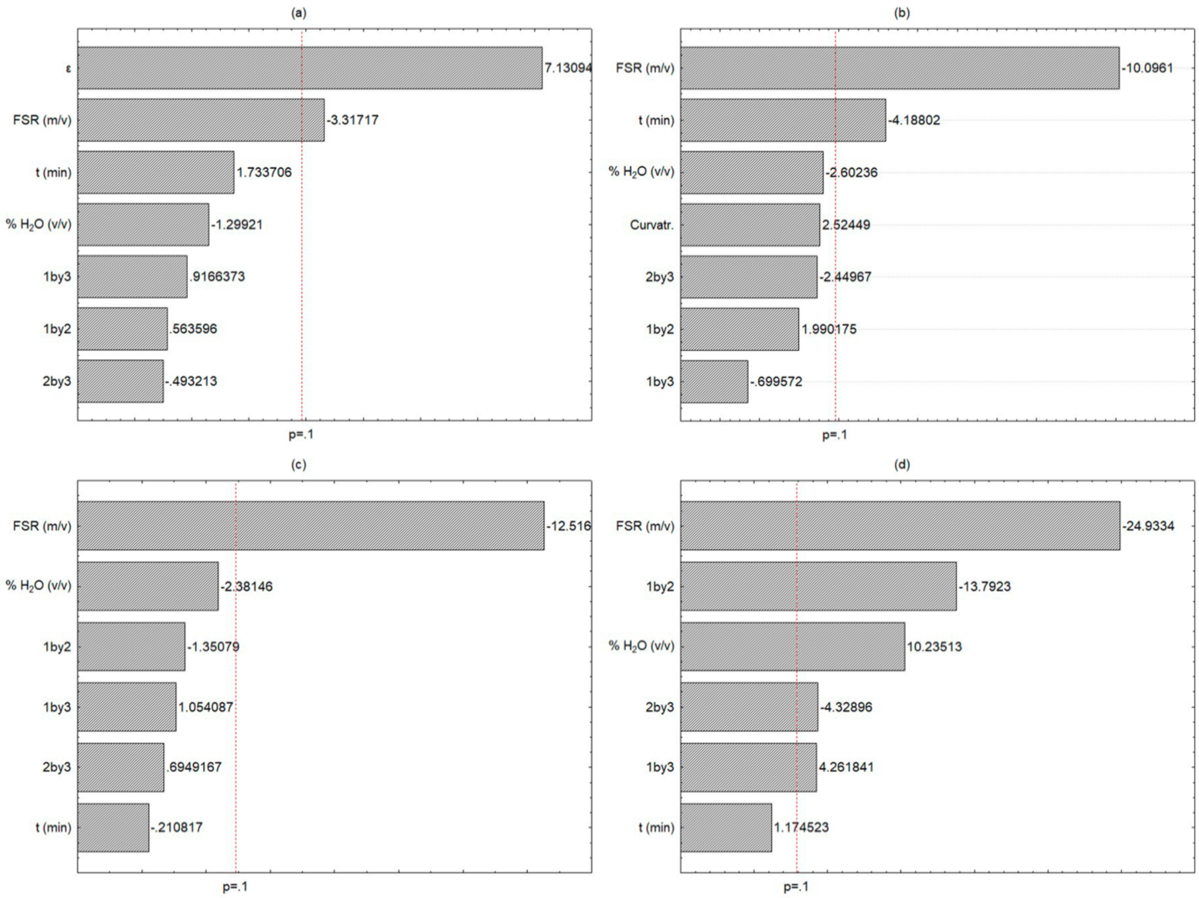

Figure S1. Pareto charts for standardized effects estimates (absolute values) of ultrasound-assisted extraction of (a) total phenolic content, (b) antioxidant activity, (c) gallic acid content and (d) quercetin content from freeze-dried cutite fruits.

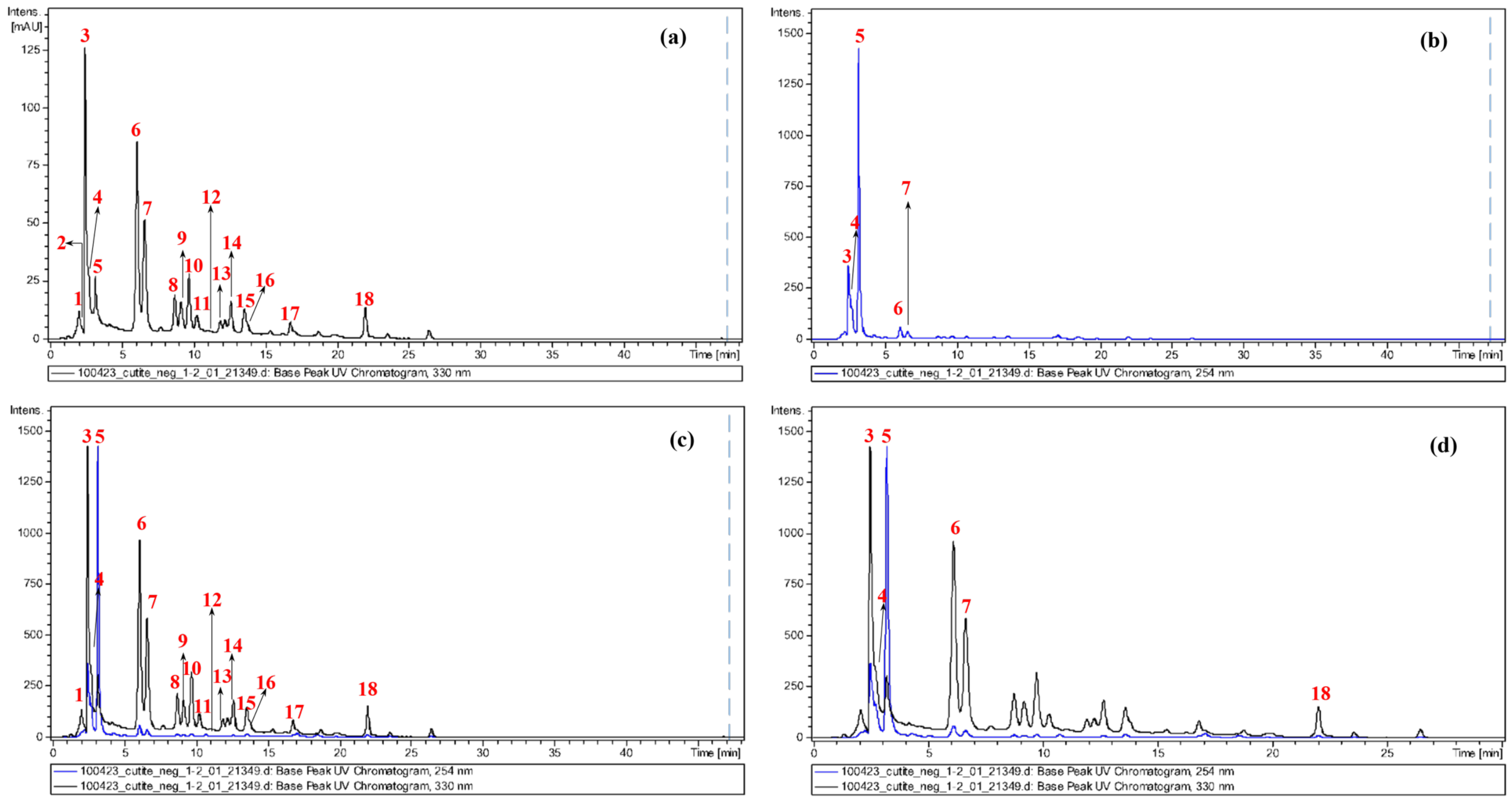

Figure S2. UV-VIS chromatogram of *P. macrophylla* extract, where (a) = UV-Vis chromatogram at 330 nm; (b) = UV-Vis chromatogram at 254 nm; (c) = UV-Vis chromatogram overlaid at 254 nm (in blue) and 330 nm (in black) wavelengths; (d) = UV-Vis chromatogram expanded.

Table S1. ANOVA for the effects of ultrasound-assisted extraction of phenolic compounds, gallic acid and quercetin and antioxidant activity from freeze dried cutite fruits with different solvents using a quadratic response surface method.

	Variation source	Sum of Squares	Degree of Freedom	Mean Square	F-value	R^2
**TPC**	Regression	18,172,228.96	7	2,596,032.71	14.5537851*	0.9714
	Residue	535,125.2675	3	178,375.089		
	Lack of fit	244.71	1	244.7094	0.0009**	
	Pure error	534,880.56	2	267,440.2790		
	Total	18,707,354.23	10			
**AA**	Regression	1377685,000	7	196812.143	27.955*	0.986
	Residue	21121,000	3	7040.333		
	Lack of fit	1862,000	1	1862.200	0.193**	
	Pure error	19259,000	2	9629.700		
	Total	1398806,000	10			
**GAC**	Regression	47,25162	6	7.87527	21.2888288*	0.9883
	Residue	1,4797	4	0.369925		
	Lack of fit	0,90969	2	0.45484	1.59592982**	
	Pure error	0,57001	2	0.285		
	Total	48,73132	10			
**QC**	Regression	1,147341	6	0.1912235	34.6184205*	0.99794516
	Residue	0,022095	4	0.00552375		
	Lack of fit	0,019692	2	0.009846	8.19816819**	
	Pure error	0,002403	2	0.001201		
	Total	1,169436	10			

$F_{0.1;\,6,4}$: 4.01; $F_{0.1;\,2,2}$: 9 (F = tabulated value from Fisher test); *significant model and **predictive model. The mathematical models were considered significant when the regression F_{values} were three times higher than $F_{tabulated}$ and as predictive when the lack of fit F_{values} were lower than the $F_{tabulated}$ value.

Table S2. Regression coefficients (RC) and its p-values for each one of the responses.

**Response**	**Term**	**RC**	***p*-value**	**Response**	**Term**	**RC**	***p*-value**
**TPC**	Mean	1433.790	0.016*	**GAC**	Mean	12.105	0.014*
	β_1	-237.546	0.323		β_1	-0.013	0.612
	β_2	-606.507	0.080*		β_2	-0.423	0.065*
	β_3	316.989	0.225		β_3	-0.070	0.342
	$\beta_{1,2}$	103.047	0.630		$\beta_{1,2}$	-0.002	0.309
	$\beta_{1,3}$	167.597	0.456		$\beta_{1,3}$	0.0008	0.402
	$\beta_{2,3}$	-90.178	0.671		$\beta_{2,3}$	0.003	0.559
	Curvature	2496.612	0,019*				
**DPPH**	Mean	876.323	0.002*	**QC**	Mean	0.363	0.0628*
	β_1	-90.288	0.121		β_1	0.014	0.0103*
	β_2	-350.278	0.0097*		β_2	0.028	0.0637*
	β_3	-145.301	0.052*		β_3	0.0016	0.7052
	$\beta_{1,2}$	69.048	0.185		$\beta_{1,2}$	-0.0014	0.005*
	$\beta_{1,3}$	-24.271	0.557		$\beta_{1,3}$	0.0002	0.051*
	$\beta_{2,3}$	-84.990	0.134		$\beta_{2,3}$	-0.0011	0.0494*
	Curvature	167.714	0.128				

β_1= %H_2O (v/v); β_2= Fruit Solvent Ratio (FSR) (w/v); β_3= time (min); $\beta_{1,2}$= interaction between %H_2O and FSR; $\beta_{1,3}$= interaction between %H_2O and time; $\beta_{2,3}$= interaction between FSR and time. *Significant effects when p-value≥0,1.
